# Understanding Experiences of First Contact Physiotherapy in General Practice: A Realist Qualitative Study

**DOI:** 10.1177/21501319251404273

**Published:** 2026-01-13

**Authors:** Hannah Stott, Alice Berry, Serena Halls, Rachel Thomas, Cathy Liddiard, Justin Jagosh, Fiona Cramp, Margaret Cupples, Paula Kersten, Zoe Anchors, Peter Williams, Heather Gage, Dave Foster, Nicola E. Walsh

**Affiliations:** 1 University of the West of England, Bristol, UK; 2 University of Bath, UK; 3 Centre for Advancement in Realist Evaluation and Synthesis, Vancouver, Canada; 4 Queen’s University Belfast, UK; 5 University of Suffolk, Ipswich, UK; 6 University of Surrey, Guildford, UK; 7 Patient Research Partner, Bristol, UK

**Keywords:** First Contact physiotherapy, realist evaluation, general practice, primary care

## Abstract

First Contact Physiotherapy Practitioners (FCPPs) are working across the UK to deliver musculoskeletal services to patients within general practice. Little is known about the impact of the model and how variation in delivery may lead to different experiences and outcomes of the service amongst patients and general practice staff. This study explored staff and patient experiences of First Contact Physiotherapy in general practice to determine what works, for whom, under what circumstances, and how. Qualitative interviews were conducted in general practice sites across Great Britain. Interviews were based on initial programme theories identified in an earlier realist review and consensus event. We conducted 80 remote interviews with practice staff and patients, and a further 3 interviews with respondents with other roles related to First Contact Physiotherapy provision. All interviews were analysed using a realist approach. Seven overarching theory areas were identified: 1. Awareness of FCPPs; 2. Communication and integration into practice; 3. FCPP approach in primary care; 4. FCPP additional qualifications; 5. Practice workload; 6. FCPP personal development; and 7. Employment models of FCPP. Three key areas were identified as integral to successful service delivery and implementation: FCPP integration; skillset and impact on resource use; and employment model. Findings from this study strengthen the evidence by providing a robust piece of empirical work about the key issues and contexts impacting successful implementation of the FCPP role into general practice, which will aid decision makers when developing First Contact Physiotherapy services.

## Introduction

Musculoskeletal (MSK) Disorders are the leading cause of disability in the UK^1,2^ accounting for 28.4 million lost workdays in 2019,^3^ costing NHS England almost £5 billion per annum,^4^ and accounting for up to 30% of all GP consultations in England.^5^ Alternative models of care which are implementable, affordable, and sustainable are needed.^6^

One model is First Contact Physiotherapy, which has been rapidly implemented across the UK. First Contact Physiotherapy Practitioners (FCPPs) are located and embedded within general practice where they assess, diagnose, and determine management plans for patients presenting with MSK disorders, without the requirement for prior GP consultation.^7^ The FCPP model was expedited by the Additional Roles Reimbursement Scheme in England which committed to providing access to FCPPs to all patients by 2024.^8^

An evaluation of FCPP provision in advance of expedited roll out, suggested short term improvements in clinical outcomes^9^ and qualitative analysis demonstrated positive perceptions of FCPPs by practice staff, and patient satisfaction.^10^ A UK-wide evaluation by our group demonstrated clinical and cost benefit compared to GP led models,^11^ but other work has recognised implementation challenges including lack of role acceptance, and difficulties with appropriate triage.^12^ Less is known about how FCPP services are implemented in different settings with variations in delivery, potentially leading to different experiences and outcomes.

The aim of this project was to gather qualitative evidence from key stakeholders to understand how embedding FCPPs within general practice across the UK, and barriers and facilitators to successful implementation.

## Methods

This realist qualitative study was embedded within a larger realist evaluation of the clinical and cost effectiveness of First Contact Physiotherapy role in primary care (NIHR ID: 16/116/03). Ethical approval was granted on 18/06/2019 (IRAS ID:261530; REC reference number: 19/NI/0108).

### Methodology

Realist methodology was used to understand how the FCPP role works within general practice given variations of the model and diversity in contexts of implementation.^13^

Realist methodology is concerned with understanding interactions between mechanisms of a programme (in this case the casual impact of introducing the FCPP within general practice), the contexts within which this happens (eg, practice location, size of team, and competencies of FCPP staff), and outcomes.^14^ Outputs are generated to provide retroductive (explanatory) insight about how the programmes works, for whom and in which circumstances.^15^

### Study Process

This study built on a preparatory rapid realist review and stakeholder engagement which highlighted key theory areas of the FCPP ‘architecture’ which likely influence the effectiveness of the service such as: (1) Practice understanding of the role; (2) Integrating the FCPP into general practice; (3) FCPP knowledge and skills; (4) Appointment structure; (5) Practice endorsement of FCPP; (6) Patient acceptability of the FCPP role; (7) Employment and management of the FCPP role; and (8) FCPP impact on practice workload and wider resource use. Initial Programme Theories (IPTs) in the form of ‘If-then’ statements were created^13^ and explored through realist interviews with study participants.

### Realist Interviews

Interviews were guided by a realist methodology expert (JJ) and topic guides were aligned to Initial Programme Theories. A realist approach to following up points of interest was used, whereby IPTs were explored, validated, and refined. This allowed the interviewer to pinpoint and summarise certain issues raised by the participant to ensure common understanding, and to clarify any causal assertions.^16^

### Participants

Realist qualitative interviews were conducted with individuals from general practice sites who had taken part in the quantitative analysis of their service. These practices were identified through a UK wide survey^17^ and supplemented by practices who were identified through the Clinical Research Network. Site selection was stratified to include all UK nations, geographical location, deprivation levels and FCPP capabilities. Full details can be found in Walsh et al^11^ Stakeholders included: (i) practice reception staff; (ii) GPs; (iii) physiotherapists; (iv) patients; and (v) practice managers. A maximum variation approach to sampling was used to ensure that sites represented an array of types of FCPP employment, clinical capabilities and demographic attributes. Selected practices also reflected important demographic variables including geographic factors (urban or rural location) and level of deprivation (high, moderate, or low).

### Recruitment

Staff stakeholders were approached at each site via a local investigator. Potential patient participants indicated if they would like to take part when completing outcome measures as part of the wider realist evaluation.^11^

Staff and patients who expressed an interest verbally consented to be contacted by the study team who emailed or posted a participant information sheet, consent form, and interview guide before booking a convenient interview time. One-to-one interviews were conducted remotely by members of the study team (HS and SH [health services researchers], and NW [academic physiotherapist]), via video or telephone call. Following each interview, immediate observations and thoughts were documented by the interviewer. Practice level interviews were undertaken from June 2020 to April 2021 under COVID-19 restrictions. Three additional interviews with musculoskeletal interface staff and FCP educational lead were undertaken in June 2022 to provide clarification on emerging theories.

### Analysis

Interviews were transcribed and anonymised. Each transcript was reviewed by 2 researchers across the team. The first reviewer read the transcript and the interviewers’ reflections and created their own journal style reflection. Following this reviewer 1 re-read the transcript and highlighted key quotes within the transcript to provide causal links in both a deductive manner (relating to existing theories) and inductively (generating new links and theories). Thinking around CMO formations was included alongside supporting quotations. Reviewer 2 read the primary review, providing additional insight, agreement or alternative interpretation. Where alternative interpretation of the data existed, this was presented as a rival theory 13 and taken to forward to explore these variations. Annotated transcripts were imported into NVIVO, and data were extracted to the theory areas of interest. These data were then used to create evidenced CMO configurations.

## Findings

In total, 24 GP practices took part with 80 interviews representing stakeholders at each site (Table 1). To gain wider system contextual information 3 additional interviews were conducted with a musculoskeletal interface clinician, interface lead, and university training lead for FCPP. Interviews were on average 29 min.

**Table 1.** Participating Practices and Interviewees.

ID	Country	FCPP category	Urban/rural	Deprivation	Interviews conducted with stakeholders	Total
01	Wales	FCPP(AQ)	Rural	High	FCPP, GP, Practice manager, Receptionist, 2 Patients	6
02	Scotland	FCPP(AQ)	Urban	Low	FCPP, Practice manager, Patient	3
03	Scotland	FCPP(AQ)	Urban	Low	Patient	1
04	Wales	FCPP(AQ)	Rural	Moderate	FCPP, Practice manager, GP, Receptionist, 4 Patients	8
05	Wales	FCPP(AQ)	Urban	Moderate	FCPP, GP	2
06	Scotland	FCPP(AQ)	Urban	Moderate	FCPP, GP, 2 Patients	4
07	England	FCPP(AQ)	Urban	Low	4 Patients	4
08	England	FCPP(AQ)	Rural	Moderate	GP, Practice manager, FCPP, 2 Patients	5
09	England	No FCP	Urban	Moderate	GP, Patient	2
10	England	FCPP(ST)	Urban	High	FCPP, 2 GPs, Practice manager, Receptionist	5
11	England	No FCP	Rural	Moderate	GP, Practice manager, Receptionist	3
12	England	No FCP	Urban	Low	GP, 2 Advanced Practitioner nurse	3
13	Wales	FCPP(ST)	Urban	High	FCP	1
14	England	No FCP	Urban	Low	Physiotherapist (non-FCPP), GP, Practice manager, Receptionist	4
15	England	FCPP(AQ)	Urban	High	FCPP, Receptionist, Patient	3
16	England	No FCP	Rural	Low	GP, Practice manager	2
17	England	FCPP(AQ)	Rural	Moderate	FCPP, GP, 2 Patients	4
18	England	FCPP(AQ)	Urban	High	Patient	1
19	England	FCPP(ST)	Urban	High	FCPP, 2 Patients	3
20	England	FCPP(ST)	Rural	Moderate	FCPP, GP, 2 Patients	4
21	England	FCPP(AQ)	Urban	High	FCPP, GP, Patient	3
22	England	FCPP(ST)	Urban	Moderate	2 FCPP, GP, Patient	4
23	England	FCPP(AQ)	Rural	Moderate	Patient	1
24	England	FCPP(ST)	Rural	Low	FCPP	1

Abbreviations: FCPP(AQ): FCPPs with expert MSK skills and additional qualifications to inject and/or prescribe; FCPP(ST), FCPPs with expert MSK skills.

IPTs generated during the rapid realist review and refined through this study were encapsulated under 3 theory areas: (1) Understanding the FCPP role and integration into healthcare teams; (2) The FCPP approach and skillset; impact on workload and resource use; and (3) Employment of the FCPP role.

### Understanding the FCPP Role and Integration Into Healthcare Teams

#### Promoting the FCPP Role to Patients

Practices emphasised how effective promotion of the FCPP role was key to making the service an ‘acceptable’ alternative to GP appointments:

From the initial outset patients were a bit wary . . . . . . Now we have a bit of a spiel. ‘The advanced physio will see you, who can assess, diagnose, can prescribe . . . ’ . . . they’re very reassured at the outset . . .’ Practice manager, 04

It was possible to build patient confidence in the FCPP skillset if the GP endorsed the role (*GP, 20)* however, there were challenges when describing the FCPP role to patients. Using terms such as ‘senior’ or ‘advanced’ highlighted the specialism of the role to encourage patient confidence, and some clinicians expressed concerns that ‘first contact’ implied patients were not allowed to return to the FCPP (*GP2, 10)* whereas many were offering follow-up appointments if deemed necessary.

#### Developing Staff Understanding of the FCPP Role

The onus was on the FCPP to communicate their role to practice staff; however, this could be challenging due to the need to balance clinical workload with these duties (*FCPP, 17*). FCPPs used a variety of techniques to improve staff understanding of their role including regular training about the role, daily reminders, staff feedback, information sheets, and videos.

When FCPPs experienced challenges communicating the role to practice staff they found GPs unnecessarily referred patients to them, leading to duplicated workload and increasing patient pathways:

I tried to indicate that needs to go onto MSK physio, not to an FCP if he’s [GP] already made the diagnosis . . . he’s more or less using me as an expert physio in-house, as opposed to an FCP and I don’t think he’ll change his mind on that easily . . .’ (FCPP, 20)

CMO: Promotion of the FCPP role amongst practice staff teams

**Table:** 

C: FCPP role scope and referral processes are new to primary care. It takes time for staff and practices to develop familiarity with the role. Patients are reluctant to consult with an FCP as ‘traditional’ models of care require an initial GP consultation to determine the most appropriate course of management. Additionally, understanding of the FCP skillset and role is limited resulting in further patient uncertainty and reticence, and staff confusion as to how the FCP is most effectively utilised. M: Providing the FCPP with time and materials to educate staff and patients about their role (resource); reception staff addressing patient reluctance and refusal to consult with FCPs through explanation and differentiation from standard GP, supported by further endorsement from GPs regarding the nature of the consultation (resource) enhances patient understanding, reassures them of the role and benefit of the FCP (response) Providing the FCPP with time and materials (resource) helps develop other staff and patient knowledge about FCPP scope, trust in the FCPP role, staff confidence to endorse the role to patients, and familiarity regarding which patients the FCPP sees (response). O: Over time FCPP caseload improves (relevance, scope of practice), which shortens the patient pathway leading to reduced GP appointment use, and improved patient and staff acceptability of the role.

#### Secondary Care and Dual Role Physiotherapists

FCPPs who worked dual roles, as an FCPP and within a local department, were often well integrated into MSK pathways outside of general practice. This knowledge of clinicians and pathways allowed them to make more targeted referrals, accelerating the patient journey. In some instances however, secondary care services were limiting referrals from FCPPs due to workload and waiting lists, and requested all referrals were sent via GP or local musculoskeletal assessment teams. This added workload to these teams and increased the patient pathway:

Not all secondary care places will take referrals from us . . . so that’s a little bit of a barrier . . . I think they think they’ll be inundated . . . I’ll write the letter, email it to the GP, who then just cuts and pastes it, so it’s essentially my referral. (FCPP, 06)

#### Integrating the FCPP Into the Practice

At some practices availability of space to physically co-locate the FCPP was problematic and FCPPs had to change rooms *(10)*, were placed in inaccessible rooms *(13)*, or rooms that were too small or lacked necessary assessment facilities *(15)*. However, co-locating FCPPs in general practice provided advantages to patients, particularly in rural areas where access to physiotherapy departments was hampered by geographical distance *(04)*. It was universally recognised that FCPPs were most effective when considered a member of the multidisciplinary practice team who provided a joined up and coherent approach to patient management:

[FCPP is] most efficient when the multi-disciplinary primary care team work together. So, . . . the triage nurse who’s been speaking with patients in the morning can hand over any patients to MSK or to GP. We very much have an open-door policy and a duty GP, so there’s always someone available for escalated discussions. (Practice Manager, 02)

FCPPs highlighted the need for formal and informal, virtual, and face-to-face opportunities for communication with staff teams, and the need to know who to approach each day for clinical discussions. These issues became more pertinent for FCPPs not employed directly by practices, as some described not being invited to meetings, or missing meetings that did not coincide with their session times. This led to a perception of less integration and disjointed working:

I don’t feel fully integrated into the practice . . . the GP practices that we cover are all part of one larger group . . . one of them that I worked at was just really friendly. Whereas, at the other practice . . . I would just kind of feel a bit more like ‘Oh they don’t know me, and I don’t know who they are (FCPP, 24)

Being able to work in a multidisciplinary way, where FCPPs had good knowledge of patient history for example, was key to successful implementation of the role:

in the other GP practice that she works in there’s a patient that has . . . spoken with the GP but then had been referred to her, but there had been a lengthy wait to see her and then COVID happened . . . that the patient, I think, had something, I’m afraid to say, sinister and . . . it wasn’t missed, it just wasn’t possibly managed and I think she [FCPP] felt that, at that practice there wasn’t a great . . . ability to speak with everyone (Practice Manager, 02)

This was the only instance where safety issues were identified, and even then, not related to skill set, rather than lack of clear communication channels. FCPPs were reported by other clinicians to work safely with patients and escalate red flags appropriately.

CMO: Factors impacting successful FCPP integration into the practice team

**Table:** 

C: Practices who work in a multidisciplinary way, with established methods of communication are able to better support the embedding of FCPPs. M: FCPPs spend more sessions within fewer practices, have allocated time for networking, and open communication avenues. They access and share clinical expertise to facilitate more informed clinical decisions. Integration allows continuous feedback to staff regarding referral management (resource), making them feel part of the team, and better integration (response). O: Shortened patient pathway and improved patient outcomes, reduced GP workload over time, Staff satisfaction, improved service efficiency, and safe service provision.

### Impact of the FCPP Approach and Skillset on Workload and Resource Use

#### The FCPP Approach

There was agreement between staff and patients that FCPP musculoskeletal expertise was at a higher level than the GP.

he’s very good diagnostically – we value that. . . . we all know our musculoskeletal stuff to an extent, but I always feel like physios . . . they know a lot more than us GPs! They’re much more thorough. They can get to the bottom of things very quickly . . . (GP, 17)

The ability to diagnose accurately and quickly was attributed to the extended appointment times FCPPs offered. Respondents reported appointment times between 15 and 30 min, with most offering 20-min appointments. Both GPs and FCPPs acknowledged that to conduct an in-depth assessment and achieve the associated improved patient management, it was key to provide FCPPs with longer appointments than the GP. This was thought to lead to reduced prescribing and appointment use over time:

We [FCPPs] get longer than the GP . . . we can allow patients to explain themselves rather than directing sessions with closed questions . . . I would see some of these people once, they see them four or five times for the same thing . . . they will check them up periodically and people are phoning in more to have painkiller reviews and things rather than addressing what’s actually going on (FCPP, 17)

The additional appointment time was well received by patients who described feeling listened to and understood by the FCPP:

I felt it was very thorough . . . the exercises she suggested I do, seemed to be very relevant . . . . I think just it was nice to not feel rushed . . . she explained what she suspected the problem was . . . she obviously took the time to show me the exercises she wanted me to do and then she got me to sort of try doing them (Patient, 06)

In general, the FCPP was perceived positively by patients. However, a minority challenged the acceptability of the FCPP approach.

I thought ‘No, I want an x-ray’ and I didn’t want to wrangle with him over my shoulder because I was in agony . . . (Patient, 01)

This issue was acknowledged by FCPPs too, who had to challenge patient expectations around imaging, whilst not increasing the likelihood of patients consulting the GP and utilising additional appointments if diagnostic expectations had not been met *(FCPP, 20).* FCPP recommendations that patients utilise exercises and self-management for MSK disorders had mixed responses from patients. If patients understood and engaged with self-management advice and had confidence that it might help, they were more likely to experience an improvement in symptoms:

I thought ‘Oh, this’ll never do any good’ because it was so painful but, in fact, it probably did, because it does feel a lot better now . . . I think the exercises have done me good (Patient, 04)

A key aspect of FCPP intervention was the offer of follow up after initial consultation. The majority offered follow up calls if needed, enabling patients to monitor their condition and feel reassured that they would receive further intervention if necessary. If this was missed, then it created uncertainty for the patient which could lead to increased GP appointment use:

He said he would call four weeks after I had my injection and I’ve not heard from him . . . that kind of disheartens you to thinking . . . ‘I might as well just go back to my GP then.’ . . . if it does not get better or it doesn’t get fixed you can’t get closure on that injury (Patient, 03)

CMO: FCPP MSK knowledge and approach is more specialised than GP

**Table:** 

C: FCPPs have specialist MSK knowledge compared to GPs and are allocated longer appointment times (20+ minutes). M: This enables the FCPP to conduct a more thorough assessment, diagnose more complicated MSK disorders, determine the necessity for further investigation, and provide instant access to specific non-pharmacological interventions (e.g., tailored exercise regimens) (resource) and condition related advice (resource). This reassures and empowers patients to take self-directed action (response) and reduces the need for GP onward referral for physiotherapy input (response). O: Improved patient outcomes and satisfaction; Staff satisfaction; fewer appointments required (in onward referral); and fewer prescriptions.

#### FCPP With Additional Qualifications and Workload

Additional qualifications of the FCPP were perceived to impact practice efficiency, being able to take on a higher proportion of patients and reducing GP workload. It was felt that FCPP injection qualifications provided a bigger reduction to GP workload than prescribing due to the time it takes to administer injections:

He [FCPP] undertakes other things as well, for example, joint injections and things so quite hands-on . . . I suppose it allows me to deal with other things. I’m not bogged down with just MSK . . . (GP, 01)

Some FCPPs took an alternative perspective to utilising injections, adopting a more conservative approach by reducing the number of injections used and using other treatments leading to improved condition management:

We’re trying to move away from just the current injection, injection, injecting tendon pains and things like that . . . it’s making sure you’ve exhausted all the conservative treatments and everything else first before we start . . . I think we had something like 60 people waiting for an injection at one point, and I cleared that list, really. I went through that list, and I probably only injected about a third of those patients in the end (FCPP, 22)

FCPPs without additional qualifications and FCPPs who experienced information technology or governance barriers to being able to administer prescriptions, had to task GPs or other clinicians to provide prescriptions, yet this was still perceived by GPs as a reduction in workload because they did not have to conduct the whole patient consultation themselves *(GP, 05)*.

At the time of interviews FCPPs were frustrated that they were not legislated to prescribe and were not able to provide patients with certification confirming whether or not they were fit to work (fit notes), which they felt would be a useful addition to their role, saving patient time and reducing GP workload. Since completing data collection for this study, legislation changed, allowing FCPPs to administer fit notes (July 2022).

CMO: FCPPs with additional qualifications alleviate workload and reduce patient pathway

**Table:** 

C: Some FCPPs have additional qualifications (e.g., injections/prescribing) meaning they can independently manage a higher proportion of MSK patients and deal with patients largely autonomously. M: Initially, more time is required from GPs (resource) up front to ensure that FCPPs receive adequate supervision/mentoring, and financial investment, to gain the skills required (response), over the longer-term this frees up GP MSK workload (reduced patient volume and FCPP supervision) (resource) to deal with other patients (response). FCPP injection skill (resource) may reduce GP workload through decreased appointments (response). FCPPs with additional pharmacological qualifications (resource) may also reduce GP workload through decreased appointments and time to sign off prescriptions (response); number of prescriptions/injections may also reduce due to FCPP ability to use other skillsets e.g., tailored exercise and self-management approaches. O: Improved patient outcomes for MSK and non-MSK patients, GP satisfaction, shorter MSK patient pathway, patient satisfaction, reduced healthcare resource use, reduced burden on secondary care appointments.

#### Managing Referral of Complex MSK Presentations and Staff Training in MSK Disorders

There was variation amongst practices in how to best manage patients with complex conditions alongside an MSK disorder. In some practices these patients were managed solely by the GP to provide a more streamlined interaction for the patient and reduce appointment use *(01, 04)*. In another practice other problems were dealt with by the GP and the patient was then referred onto the FCPP for consultation regarding their MSK disorder, thus freeing up GP time *(20)*. Another practice made a pragmatic decision to share MSK workload between FCPPs and GPs because there was too little FCPP time to manage all patients with MSK disorders, and GPs liked this type of work *(06)*. One site reported an advantage to bringing together the expertise of the FCPP and GP to managing complexity *(21)*:

My colleague GP and myself, we both very closely work and review at the same time pain medication also, especially chronic conditions because we tend to see in this practice, repeat patients . . . We wonder what’s going on you know? Whether we diagnosed properly and then, if needed, we do further investigations and find the problem and right medication and right exercise advice (FCPP, 21)

It was not always clear whether patients who had a chronic MSK disorder and were under GP care could be referred to the FCPP as they fell outside of the ‘first contact’ criteria. Some practices referred these patients to the FCPP to ensure their lists were full as the service developed, or to utilise FCPP expertise to improve patient care:

If a patient comes to us first, strictly speaking we’re not supposed to send them onto [name] because that sort of defeats the object. But there is a bit of flexibility with it. We have discussed patients with him that we’re concerned about, and he’s arranged to see them. I don’t know whether that’s actually allowed, strictly speaking, but it definitely helps . . . our problem is, NHS physios, the waiting list for that at the hospital for here is just, I don’t know, I dread to think how many months or weeks it is . . . he’s picking stuff off the list so that’s reducing our workload, which we really need at the moment because we’re drowning a bit . . . (GP, 17)

Whilst FCPPs undoubtedly provided advantages to patient care and GP workload, it was also important to ensure other staff could access some of these patients to retain MSK skillsets and develop experience in this area:

My concern is for our GP trainees. I am trying to make sure that they get some musculoskeletal stuff booked in, or if they get stuff booked in that they don’t just kind of push it on to the FCP . . . I remember my training, we did an awful lot of joint examination and that sort of thing at university but, certainly, the trainees we’ve got now I think they’re quite lacking confidence in terms of musculoskeletal problems. (GP, 20)

FCPPs were considered an important MSK training resource in general practice. Practice staff utilised FCPPs for clinical discussions to develop their MSK diagnostic and patient management skills *(19, 21-22)*, for joint appointments *(21)* and to provide training in weekly clinical meetings *(6)*.

Yesterday I said ‘Look, if you have somebody that you’ve seen, why don’t you book a double appointment – one for yourself, one for the FCP and watch how they would assess that patient?’ That way you will learn for yourself what a professional assessment from a physiotherapist would be like and you can use that to assess your own skillset. (GP, 14)

CMO: FCPPs can provide new insight into complex MSK presentations with support from the GP

**Table:** 

C: The practice adopts a flexible approach to FCPP referral and refers patients who have previously seen a GP for their MSK disorder without resolution or diagnostic uncertainty. M: GP-FCPP review or discussion of complex MSK patients in conjunction with a referral for a thorough assessment by the FCPP (resource) provides new ideas for patient management which may rely less on medication (response). O: Improved patient care and outcomes, reduced prescriptions, GP upskilling and fewer referrals to onward physiotherapy or unnecessary investigations.

### FCPP Employment

#### Recruiting Experienced FCPPs to the Role

Practices highlighted the importance of recruiting FCPPs with a specific level of training and experience to embed the role successfully and safely within a general practice population. Witnessing FCPPs manage MSK patients successfully enabled GP and other practice staff to build trust in the FCPP role and safely delegate patients to them, thereby relieving the GP of MSK workload:

Our experience with the fully funded roles, the ARRS roles . . . is that it’s very, very much dependent on the person who’s in role . . . they are taking risks, they’re taking a responsibility and that ability to manage that risk and responsibility is all down to experience and their skills . . . the GMC rules are quite clear on that, is that when you’re delegating responsibilities you have a responsibility to make sure that they’ve got the ability to do that work for you, because you’re still held responsible for their mistakes. There is a risk involved in that and protocol and clinical governance, document and contracts only go so far. So, it’s important that we get the right person (GP, 14)

However, interviewed staff noted that there were not enough experienced FCPPs available for them to recruit into general practice. Respondents were concerned that they were pulling physiotherapy staff out of secondary care posts, and that staff coming into general practice would require additional training and mentoring to develop their competencies:

If FCPs [are] employed without past experience then they will find the role more challenging – mentoring maybe more important for these . . . we have an FCP, she’s like a Band 6 or something, when I had a one-to-one with her, she was saying she was not that confident seeing all the ankles and foot injuries (FCPP, 15)

#### FCPP Training, Supervision, and Support

The impact of recruiting less experienced FCPPs was the requirement for additional time for training and development, supervision, daily debriefs, and training competency sign off. Less experienced FCPPs described how important clinical discussions were to maintaining patient safety *(19-20)*, and the time it took to have these conversations impacted on their caseloads which could increase stress levels. It was felt that the pressure put on FCPPs more generally impacted their health and willingness to remain in the role.

it’s finding time to do the other things . . . we have training time as part of our role and I think that needs to increase a bit more . . . you can’t just do clinical – I think you would burn out . . . I think there should be perhaps 70/30 or even 60/40 (FCPP, 6)

Similarly, FCPPs reported a lack of time to complete the advanced practice roadmap where this was a requirement of their role:

These guys have had their accreditation coursework to do, a lot of them are doing their injection courses because that’s become a must, they’re trying to do their roles, they’re trying to see their patients, they’re trying to keep up to date with their admin, and they’re exhausted. (Interface clinician)

Supervision was fundamental to FCPP development, confidence, and safe practice. Some FCPPs reported that supervision input was inadequate, or supervisors were not clearly identified or accessible.

I’m new into post and sometimes I think just having a little bit more support . . . I think that’s very important . . . I’ve not had any supervision from any of the GPs at all. I’m not aware that anyone has gone through any of the patients I’ve seen to check that they’re happy with my handling of the patients . . . a couple of times when I’ve had a very complex patient, I would have valued somebody just to sit and chat through. When I very first started, the first week, I said, ‘Who do I have time with to chat over patients?’ and one of the GPs said, ‘Why would you be needing to do that?’ . . . (FCPP, 20)

FCPPs valued connections with colleagues in general practice and highlighted that the role could be clinically isolating at times, particularly in posts without other FCPPs nearby. To alleviate this many FCPPs accessed other avenues of support beyond general practice, such as FCPP or physiotherapy colleagues in other primary or secondary care roles.

CMO: FCPP peer support

**Table:** 

C: FCPPs are new to primary care and feel isolated compared to working in busy outpatient departments. Rural locations, part time and remote working, and Primary Care Network employment models (which may inhibit integration into one practice team) make it difficult for FCPPs to gain face-to-face support from their peers. Having clinical discussions with other MSK experts is important for confidence and development in the role. M: FCPPs utilise clinical networks and peer support within and outside of general practice using face-to-face and virtual communication (resource). This improves clinical decision making and a feeling of clinical support which reduces feelings of isolation (response) and enhances safety-netting (response). O: Improved patient outcomes and safety, development of staff skillset and staff satisfaction.

#### FCPP Employment Models

FCPPs described variation in their experience based on how they were employed. Some were employed through a central provider (eg, NHS community physiotherapy services), others were employed directly by a single practice or primary care network. Being employed directly by a single practice or network provided FCPPs with clarity about who held responsibility for supporting their role.

I’m employed by the practice . . . they are now my employer, and so there’s a responsibility there. I’m part of the team and I’m integrated into the team and my training is done in-house. (FCPP 22)

FCPPs employed under a more centralised, main provider structure experienced barriers in tailoring aspects of their employment to meet individual needs. For example, accessing laptops for home working or negotiating leave.

So, I actually ended up working from home, but it was ‘who is responsible for setting that up and developing it between the Trust and the PCN and who’s paid for what and that’s the business end of it’? That I just don’t want to be involved with!. . . I was pretty much stuck in the middle of it all . . .’ (FCPP, 08).

For many, the centralised employment model (ie, employment through main physiotherapy provider) afforded opportunities for larger groups of FCPPs to connect and be part of an FCPP team rather than working in silos at individual practices. This led to more training opportunities and autonomy to develop the role amongst themselves:

We’re managed centrally . . . so we’re not employed by the GPs, we’re managed by the health and care social partnership . . . we all work very differently depending on where we work, but our clinical governance and our CPD are run more centrally . . . we can sort out the CPD and all the sort of training issues amongst ourselves rather than have to rely on an individual practice. So, we’ve got that support network (FCPP, 06)

Similarly, there could be advantages for practices of not having to negotiate individual contracts and benefiting from the FCPP skillset without the burden of role governance.

We’ve got our physio through the [provider name] team . . . So, all that was sorted out between those really, the SLA [service level agreement] and what their salary would be . . . probably a bit of negotiation went on. You know, how many hours he could work for us. (Practice Manager, 01)

CMO: Employment models and FCPP experience

**Table:** 

C: FCPP provision to practices through a provider model delivers a more consistent service for both practitioners and GP practice. M: FCPPs continue to be employed on their substantive contracted terms and conditions via a central provider, have access to peer support networks and professional development opportunities and may work within a rotational model that still exposes them to traditional physiotherapy department placements (resources). For the practice, this provides a more stable service as the FCPP employment, professional development and performance is managed centrally (resource). O: This enables a more secure employment opportunity for FCPPs, professional support and improves job satisfaction (response). For the practice it improves service effectiveness, and knowledge of the system.

The biggest issue highlighted by FCPPs of working under a centralised employer, and therefore covering several practices, was the difficulty in getting to know teams, processes, and I.T. systems across a variety of workplaces. FCPPs experienced a steep learning curve on entry to the role; learning new processes was time-consuming and there was a risk of mistakes during this period impacting patient care. The importance of standardised systems between practices was highlighted by 1 GP.

we got some funding from NHS England through something called Quick Start and so, all the practices have been working together to change all of their templates so that we all use exactly the same template . . . And, so, it’s the same for the physio, you know, if they’ve not actually booked the patient in for a follow-up and they’ve just put a code on thinking that somebody in an office somewhere is going to call back and it’s not a code that that particular practice search on, you have a problem. (GP, 16)

CMO: Standardising systems across practices

**Table:** 

C: FCPPs are required to work across multiple sites with different IT systems or processes in place which may restrict access for staff and can be confusing and time-consuming. This can lead to mistakes being made which impact patient care/safety and is inefficient for staff. M: Governance and practice systems can be managed centrally to develop standardised systems (resource), which will be quicker and easier for staff to utilise across different locations (response) and ensure that FCPPs have access to all patient information. O: Improved consistency of service, staff satisfaction, improved patient safety, and better clinical decision making.

#### Service Design and Implementation

Safe, effective, and efficient FCPP service delivery requires appropriate planning and support, and the right skillset and experience level. Data highlighted, that because of demand, staff have moved into roles without the necessary patient ‘mileage’ and skill level which has placed them in a potentially precarious and unsafe position.

I think it was rushed. . . I felt it was implemented very incorrectly. I think a lot of the FCPP staff have been hung out to dry a little bit. I think a lot of the FCPP staff aren’t qualified enough to do that role. (Interface Clinician)

There was also some thought that the full potential of the role had not been realised as FCPPs were embedded within a system that was overwhelmed. Respondents emphasised the potential for FCPP to bring about additional benefits if they moved away from the traditional throughput model in general practice. Spending time doing joint consultations would lead to upskilling of themselves and other professionals, but the thought was the system was not set up for this given the relentless demand for appointments:

It’s all about numbers and getting through patients and I suppose that comes back to the culture of primary care and is that culture around that throughput or is it around the quality and learning and the development of staff? FCPPs could be brilliant if we have the right FCPP in and they were doing a lot more of those joint consultations and that learning and case discussions. I think they could really improve care for patients . . . it was an opportunity to put physios in and to change some of that culture and to take some of that pressure and just to slow things down to improve on the quality of things rather than being on that treadmill where they’re just trying to get through numbers . . . (Interface Lead)

Respondents that worked in other parts of the MSK pathway were concerned about the impact the service was having on other parts of the system. Experienced staff moving from physiotherapy departments lead to lack of support for more junior staff elsewhere:

We’ve taken all of the good Band 6 and Band 7 more experienced physios out of department and into FCPP, so now the Band 5s are struggling, which is putting more pressure on the 8s that are around in departments and that are in the hospital, like myself. It’s upped my email and telephone workload massively (Interface Clinician)

Equally there was recognition that there were insufficient staff available to fill the new FCPP roles without impacting staffing elsewhere:

You needed 20 or 30 new physios to do the FCP and keep all those physios in department, because all you’re doing is robbing Peter to pay Paul and that, for me, is where the waiting lists are going up. (Interface Clinician)

CMO: Service Design and Implementation

**Table:** 

C: The demand for staff to fill FCPP roles means that either senior staff are migrating from department-based physiotherapy services into FCPP; or more junior, less experienced staff are taking up FCPP roles. This reduces the senior, experienced skill set in physiotherapy departments and/or places staff with less experience in frontline roles. M: Creating a system whereby junior staff are supported to develop FCPP skills within their departmental roles and exposed to FCPP alongside more senior staff (resource) would provide opportunity for role development and preparation (response). O: This would create a more structured pathway for career progression allowing staff to gain FCPP specific skills earlier within their careers enabling better preparation into FCPP roles; and develop a workforce ready to move into FCPP roles without detriment to department-based services.

## Discussion

This realist qualitative study explored patient and staff experiences of First Contact Physiotherapy in primary care, its impact on general practice, and the factors that impact successful implementation. Interview data were used to refine initial programme theories identified in an earlier realist review and consensus event with patients and healthcare professionals. The findings suggest the service was well-received, supporting previous evidence regarding the value of FCPPs in general practice.^9,18^ Three key areas were identified as integral to successful service delivery and implementation: FCPP integration; skillset and impact on resource use; and employment model.

The extent to which the FCPP is integrated into the team was considered vital, to ensure role clarity. Typically, the responsibility to communicate the scope of the role fell to the FCPP, which was difficult if they were covering multiple sites. An earlier study also reported lack of clarity leading to inappropriate referrals and resource use^19^ suggesting that insufficient improvement has been made; further supported in a recent study,^20^ investigating FCPP implementation in the Welsh health service.

Working across multiple sites, resulting from a centralised provider model, often made it difficult for FCPPs to become embedded within a practice team. Previous work has recognised the importance that FCPPs place on feeling valued, and working within a supportive, collaborative environment.^19^ Whilst practice-based support was important, the professional supportive network was also vital to FCPPs. Given the lone working nature of the role, potential for loneliness and exposure to independent diagnosis and management, FCPPs sought support from a network of physiotherapists. FCPPs have also been found to value the opportunity to contact colleagues for support with decision making when diagnostic uncertainties arise.^21^

This support was associated with the FCPP employment model. Those that were centrally employed, and therefore embedded within a larger physiotherapy service, were able to access wider support networks. This is supported by the Chartered Society of Physiotherapy (CSP) who recognise the benefits of different employment models, but suggest ‘on balance, they recommend that existing providers of NHS services employ FCPs’,^7,22^ because of the links it creates across the MSK pathway and the provision of a consistent service. Within the current study some respondents recognised the benefits of other employment models. However, substantive employment via the practice or Primary Care Network led to some FCPPs feeling more embedded within the practice.

Irrespective of employment model or embeddedness, the individual skillset and competencies of the FCPP and the model of assessment impacted the service, bringing increased MSK expertise into the practice team. The additional time afforded to FCPP appointments (most frequently 20 min duration) permitted thorough assessment of biopsychosocial issues, diagnosis, provision of immediate tailored advice, prescription where appropriate and onward referral where necessary. This is in line with CSP recommendations which recognise the necessity of additional administrative time to support the role.^7^

Whilst all FCPPs were expert musculoskeletal practitioners, they had different capabilities regarding pharmacological interventions. Some were able to inject and/or prescribe medications whilst others did not have these additional qualifications. Early implementation of the role saw many FCPPs with these competencies, and in Northern Ireland this remains a requirement for the role,^23^ but as FCCP recruitment numbers increased fewer staff had these extended skillsets. Other research has demonstrated that additional qualifications are unnecessary for FCPPs to successfully fulfil the role with no clinically significant difference in patient outcomes compared to those who cannot inject and/or prescribe.^11^ As we and others noted,^24^ FCPPs have recognised a benefit of these skills is the ability to deprescribe pharmacological interventions or offer alternative strategies rather than steroid injections, thus positively impacting resource use.

As extended roll out of FCPP continues across the UK, and in particular under the Additional Roles Reimbursement Scheme (ARRS) in England, the impact on service provision remains unclear.^25^ Demand has meant that less experienced physiotherapists are moving into these roles, and there is a likelihood that they will require additional support and supervision as they build confidence and experience to ensure quality and safety are not compromised. Some of the demand issue however may be offset resulting from greater flexibility in how ARRS funds can be used within the new GP contract. Funds can now be allocated to a wider range of staff including newly qualified GPs,^26^ as well as funding allocation based on local needs rather than capped numbers. This in itself may result in a fundamental change to skill-mix in primary care, and it remains to be seen if recruitment of more GPs may be prioritised over other staff.^27^ Further uncertainties exist regarding how the workforce will align to the ambitions of the 10 Year Health Plan in England.^28^ The intention to develop advanced practice models (including allied health professionals) in neighbourhood settings may result in fewer but higher qualified FCPPs within practice, but this remains speculative until further details are provided in the much awaited 10 Year Workforce Plan.^29^

A limitation of this study is that it was conducted when the First Contact Physiotherapy service was in a period of rapid change. New guidance was released to support implementation of the role,^22^ practice employment structures were rapidly shifting as an increasing number of allied health professionals were recruited under the Additional Roles Reimbursement Scheme,^8^ and the COVID-19 pandemic created a sudden shift in the way that clinical work was delivered in general practice. Whilst these contextual factors may have impacted perceptions and heightened challenges, we believe the issues raised are enduring and therefore relevant to current and future service delivery. Indeed, a strength of the study was that it was carried out across sites in Great Britain and explored the realities of the role during its evolution and therefore its findings have wider relevance as further staff are employed into FCPP roles. The quantitative aspect of this work did include Northern Ireland, but we were unable to recruit interview respondents from this nation.

Findings from this study highlight the importance of both patients and other members of the practice team fully understanding the role of the FCP. Success was also reliant on ensuring FCP staff being fully integrated into the team, which was in part reliant on employment models and time spent within the practice. The role of the FCP was valued but there was some concern that GP staff and trainees may become deskilled in managing these conditions. These aspects should be considered to support continued implementation of the role.
